# Breeding scheme and maternal small RNAs affect the efficiency of transgenerational inheritance of a paramutation in mice

**DOI:** 10.1038/srep09266

**Published:** 2015-03-18

**Authors:** Shuiqiao Yuan, Daniel Oliver, Andrew Schuster, Huili Zheng, Wei Yan

**Affiliations:** 1 Department of Physiology and Cell Biology, University of Nevada School of Medicine, Reno, NV 89557, USA; 2 Department of Biology, University of Nevada, Reno, NV 89557, USA

## Abstract

Paramutations result from interactions between two alleles at a single locus, whereby one induces a heritable change in the other. Although common in plants, paramutations are rarely studied in animals. Here, we report a new paramutation mouse model, in which the paramutant allele was induced by an insertional mutation and displayed the “white-tail-tip” (WTT) phenotype. The paramutation phenotype could be transmitted across multiple generations, and the breeding scheme (intercrossing *vs.* outcrossing) drastically affected the transmission efficiency. Paternal (i.e., sperm-borne) RNAs isolated from paramutant mice could induce the paramutation phenotype, which, however, failed to be transmitted to subsequent generations. Maternal miRNAs and piRNAs appeared to have an inhibitory effect on the efficiency of germline transmission of the paramutation. This paramutation mouse model represents an important tool for dissecting the underlying mechanism, which should be applicable to the phenomenon of epigenetic transgenerational inheritance (ETI) in general. Mechanistic insights of ETI will help us understand how organisms establish new heritable epigenetic states during development, or in times of environmental or nutritional stress.

Epimutation refers to an epigenetic change that causes a phenotype due to alterations in gene expression^1^. Paramutation is essentially a special type of epimutation, which is induced by a mutant allele in the other allele of the same gene^2^,^3^. The allele inducing the changes is called the paramutagenic allele, whereas the epigenetically altered homologous allele is termed the paramutant allele^2^,^4^. A paramutant allele leads to altered gene expression profiles, often associated with a phenotype. Consequently, offspring that inherit the paramutant allele may display the phenotype in the absence of the paramutagenic allele. For example, paramutation can lead to siblings that have the exact same genomic sequences, but display drastically different phenotypes^4^,^5^,^6^,^7^.

Paramutation was first reported in plants (e.g., pea and maize), and subsequently in mammals (e.g., mice)^8^,^9^,^10^,^11^,^12^. Paramutations are meiotically stable and inherited in the absence of the inducing (i.e., paramutagenic) alleles, thus representing a non-Mendelian inheritance^2^,^3^,^5^,^6^. Although more and more paramutation cases have been reported, the underlying mechanism remains largely unknown^6^,^10^,^11^. In maize, the induction of paramutations appears to be mediated by small RNAs, as evidenced by the requirement for an RNA-directed RNA polymerase, Mop1^3^,^7^. In mice, RNAs have been implicated in paramutation induction because injection of sperm or brain total RNAs, isolated from heterozygous males, can induce certain paramutation phenotypes when injected into naïve zygotes^6^,^12^. More recently, it was reported that *Dnmt2*, which encodes a methyltransferase that mostly methylates RNA, especially tRNAs, in mammals, is required for both the *Kit^LacZ^*-induced and miR-124-induced *Sox9* paramutations^13^,^14^, suggesting RNA methylation may be an essential step for paramutation establishment and/or transmission.

Epidemiological studies in humans and genetic studies in animals and plants have suggested that epigenetic information can be inherited across multiple generations^15^,^16^,^17^. This phenomenon is termed “Epigenetic Transgenerational Inheritance” (ETI). ETI has recently been defined as the “germline (sperm or egg) transmission of epigenetic information between generations in the absence of direct environmental exposures or genetic manipulations”^18^. Among reported cases of ETI in mammals, the majority are induced by environmental factors, including environmental toxicants [e.g. agricultural fungicide vinclozolin^19^, plastic additive bisphenol A^20^, pesticide methoxychlor^21^, dioxin^22^, di-(2-ethylhexyl) phthalate^23^, dichlorodiphenyltrichloroethane^24^, and hydrocarbons^25^], and poor nutritional conditions^26^,^27^,^28^. The transgenerational inheritance of paramutations is well documented in plants^4^,^7^, but in animals, there is only one case in which the paramutation phenotype is transmitted for three generations^6^,^10^,^11^.

ETI is contradictory to the established dogma pertaining to developmental global epigenetic reprogramming events, which occur during preimplantation embryogenesis and primordial germ cell (PGC) development in the fetal gonads^29^,^30^. It is believed that during the two waves of global reprogramming, epigenetic alterations gained during the lifetime of an individual, are erased and reset, thus preventing potential epimutations from being transmitted to subsequent generations. However, subsequent studies have demonstrated that neither of the two reprogramming events is complete, because many genomic loci, e.g., imprinted loci and retrotransposons (IAPs), appear to be resistant to the reprogramming events^29^. The fact that many environmentally induced epimutations appear to be transmitted across multiple generations suggests that some unknown mechanisms exist to protect those epimutations from being corrected by the global epigenetic reprogramming^16^,^18^,^29^.

The *Kit* locus has been found to be susceptible to paramutations^5^,^12^. An earlier study reported that an insertional mutation in one *Kit* allele (a LacZ gene cassette inserted into exon 1 of the *Kit* gene) caused altered *Kit* expression from the other allele, leading to a “white-tail-tip” (WTT) phenotype in genetically wild type (WT) progeny^12^. Interestingly, direct injection of RNAs isolated from *Kit^+/LacZ^* somatic tissues, or sperm, into WT zygotes, which were derived from parents completely unrelated to the paramutation family, could induce the WTT phenotype, suggesting RNAs are involved in the formation of *Kit^LacZ^* paramutation^12^. Subsequently, it was reported that microinjection of miR-1 and miR-124 into WT zygotes induced paramutation-like effects on *Cdk9* and *Sox9*, leading to cardiac hypertrophy and embryonic overgrowth, respectively^10^,^11^. These phenotypes appear to be transmissible through either the male or female germline for three generations^6^,^10^,^11^. These paramutation mouse models not only demonstrate the existence of non-Mendelian inheritance in mammals, but also serve as an excellent tool for studying the underlying mechanism.

We report, here, another paramutation in the *Kit* locus in mice, which was induced by an insertion of a copGFP gene cassette into the start codon in exon 1 of the *Kit* gene. We show, here, that the WTT phenotype, although non-specific to the *Kit^copGFP^* –induced paramutation, can be used to track the paramutation, because the incidence of this phenotype is far greater than the baseline incidence in regular laboratory C57BL/6J mouse colonies. We also demonstrate that the *Kit^copGFP^* –induced paramutation could be transmitted through either the male or the female germline. More interestingly, we demonstrated that this *Kit* paramutation could be corrected in 3–4 generations if an outcrossing scheme was used, whereas the paramutation phenotype persisted for many more, if not infinite, generations if WT mice with the paramutation phenotype were intercrossed. Moreover, we found that both paternal (sperm-borne) and maternal (oocyte) RNAs could induce the WTT phenotype, but the RNA-induced WTT phenotype failed to be transmitted through the germline.

## Results

### An insertional mutation in *Kit* locus induces a paramutation phenotype

**Figure 1:**
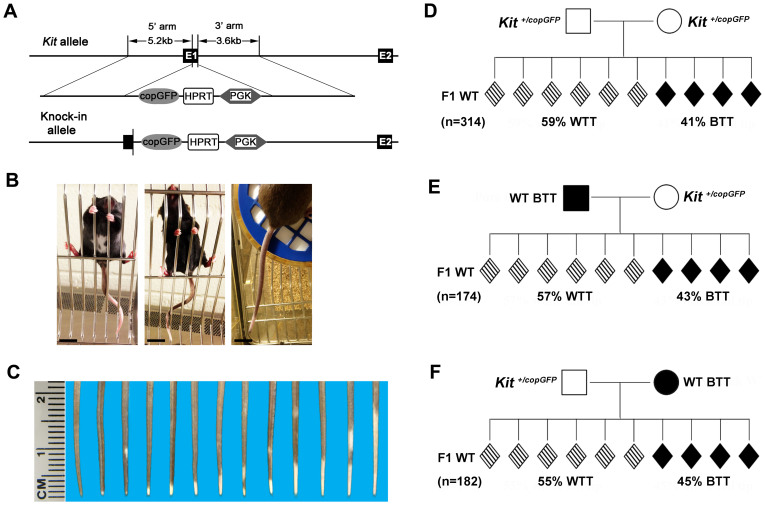
A paramutation displaying the “white-tail-tip” phenotype induced by an insertional mutation in *Kit* locus in mice. (A) Generation of a knock-in allele, which contains copGFP, HPRT and PGK gene cassettes, located immediately downstream of the start codon in exon 1 of *Kit* gene. (B) *Kit^+/copGFP^* mice display white tail tips (WTTs), white bellies and white paws (left panel), whereas a proportion of the wild-type F1 offspring derived from heterozygous parents show white tail tips (middle panel). Some wild-type129Sv/Ev mice in our colony display white tail tips (right panel). Scale bar = 1 cm. (C) Various types of white tail tip (WTT) phenotype in both the general wild-type C57BL/6J mouse colonies and the *Kit* paramutant families. (D) Penetrance of the WTT phenotype in F1 offspring of heterozygous parents. (E) Penetrance of the WTT phenotype in F1 offspring from heterozygous mothers. (F) Penetrance of the WTT phenotype in F1 offspring from heterozygous fathers. “n” denotes the total number of F1 offspring observed in each of the three mating schemes (D–F).

We previously generated a knock-in mouse line, in which a DNA fragment containing *copGFP* (from the copepod *Pontellina plumata*), an Hprt-PGK cassette, and the SV40 polyA signal, was inserted in-frame, immediately downstream of the start codon in exon 1 (Fig. 1A)^31^. The knock-in allele has been officially named *Kit^tm1(copGFP)Rosan^*. For simplicity, we called it *Kit^copGFP^* hereafter. The *Kit^copGFP^* allele is null, as *Kit^copGFP/copGFP^* are not viable and the heterozygotes (*Kit^+/copGFP^*) display white tail tips, white bellies, and white paws (Fig. 1B, left panel) in either 129Sv/Ev: C57BL/6J hybrid, or pure C57BL/6J background, with 100% penetrance. This line underwent >10 generations of backcrossing onto the C57BL/6J background when the present study was conducted.

Distribution of the *Kit^copGFP^* allele among offspring derived from heterozygous breeding pairs followed the Mendelian ratio^31^. However, we observed that ~60% of the genotypically WT progeny derived from the heterozygous breeding pairs (*Kit^+/copGFP^ × Kit^+/copGFP^*) displayed WTT with various patterns (Fig. 1C, D). Unlike *Kit^+/copGFP^* mice, these WT mice showed neither white bellies nor white paws (Fig. 1B, middle panel). The WTT phenotype is similar to that reported in *Kit^tm1Alf^*-induced paramutant mice^12^. To determine whether the WTT phenotype could be diluted through outbreeding, we then set up breeding pairs between heterozygous (*Kit^+/copGFP^*, called HET hereafter for simplicity) and pure black WT C57BL/6J mice (i.e., WT with black tail tip, called WT BTT hereafter) (Fig. 1E, F). Similar to the heterozygous breeding pairs, ~55–57% of the F1 WT progeny displayed WTTs, and these mice are called 1^st^ WT WTT hereafter for simplicity.

### The “white-tail-tip” phenotype is not unique to the *Kit* paramutation family

**Figure 2:**
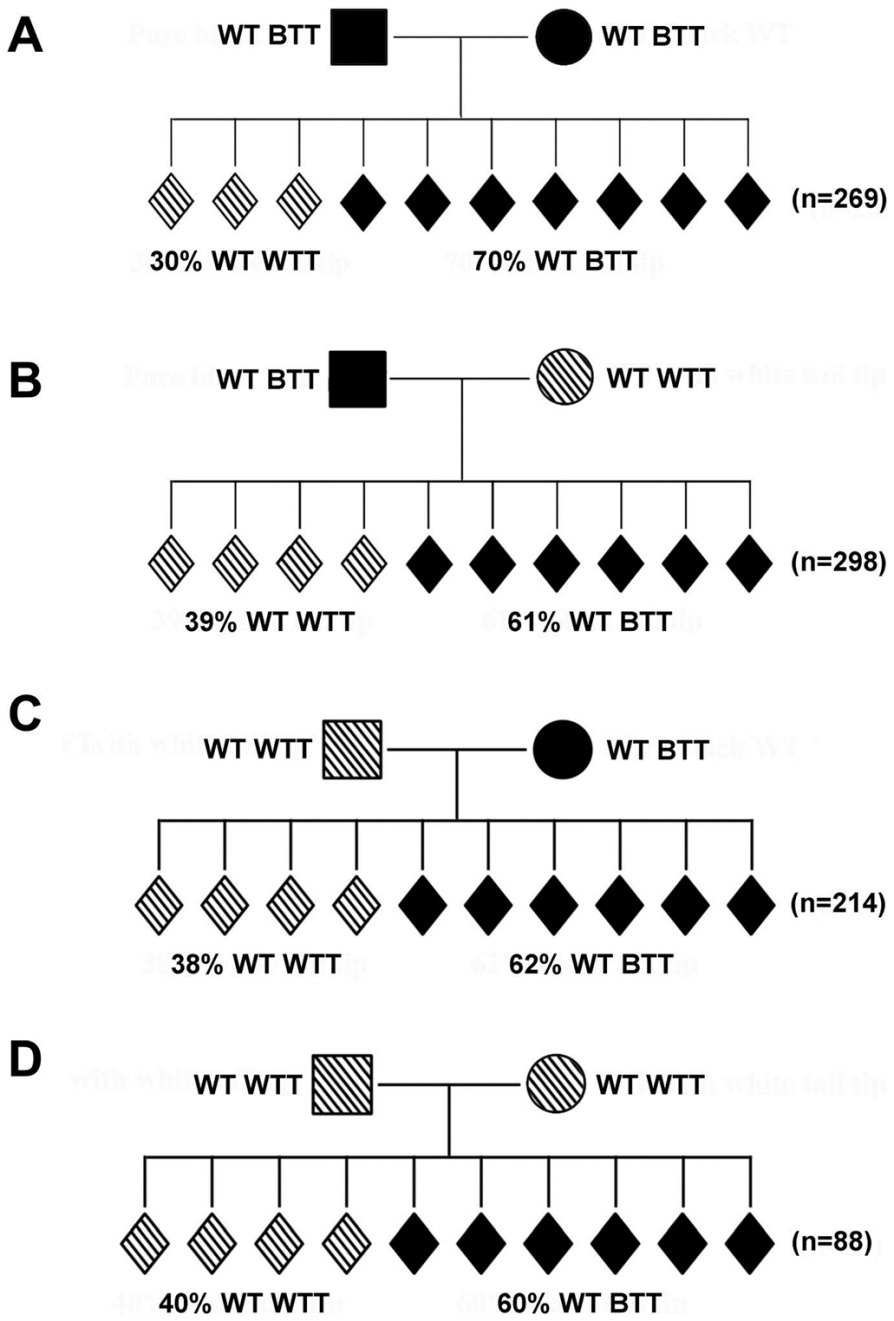
The “white-tail-tip” phenotype is not unique to *Kit* paramutant mice, as demonstrated by different breeding schemes using normal wild-type C57BL/6J mice. (A) Incidence of the “white-tail-tip” (WTT) phenotype among offspring form WT BTT parents. (B) Incidence of the WTT phenotype among offspring derived from WT BTT fathers and WT WTT mothers. (C) Incidence of the WTT phenotype among offspring derived from WT WTT fathers and WT BTT mothers. (D) Incidence of the WTT phenotype among offspring derived from WT WTT parents. “n” denotes the total number of offspring observed in each of the four mating schemes (A–D).

The WTT phenotype in *Kit^tm1Alf^*-induced paramutant mice has been questioned because normal WT laboratory mice of different strains (including C57BL/6J) display WTTs^32^. Indeed, we noticed that many of our pure WT mice, which were on either C57BL/6J or 129/SvEv (Fig. 1B, right panel) background and were totally unrelated to the *Kit^copGFP^* line, also displayed WTTs albeit at a much lower incidence. To determine the baseline incidence of the WTT phenotype among WT C57BL/6J mice, we set up four types of breeding pairs between WT males and females that were completely unrelated to the *Kit^copGFP^* line, including WT BTT males mated with WT BTT (Fig. 2A) or WT WTT (Fig. 2B) females, and WT WTT males mated with WT BTT (Fig. 2C) or WT WTT (Fig. 2D) females. ~30% of the F1 WT progeny produced by WT BTT mating pairs displayed WTTs (Fig. 2A), whereas WTTs were observed in ~38–40% of the F1 WT offspring derived from the breeding pairs with one of the parents that were WTT-positive (Fig. 2B–D). These data suggest that ~30–40% of WT laboratory C57BL/6J mice display WTTs.

Although the WTT phenotype is not specific to *Kit* paramutation, we did observe that ~55–60% of the progeny derived from one or both heterozygous parents (*Kit^copGF^*) displayed this phenotype (Fig. 1D–F), and the incidence of the WTT phenotype was significantly greater than the baseline levels (55–59% *vs.* 30–40%, χ^2^ test, p < 0.01). Thus, the WTT phenotype represents a convenient and reliable readout for tracking the *Kit* paramutation phenotype, as long as the baseline levels in the general population are taken into consideration.

### Transgenerational inheritance of the *Kit* paramutation

**Figure 3:**
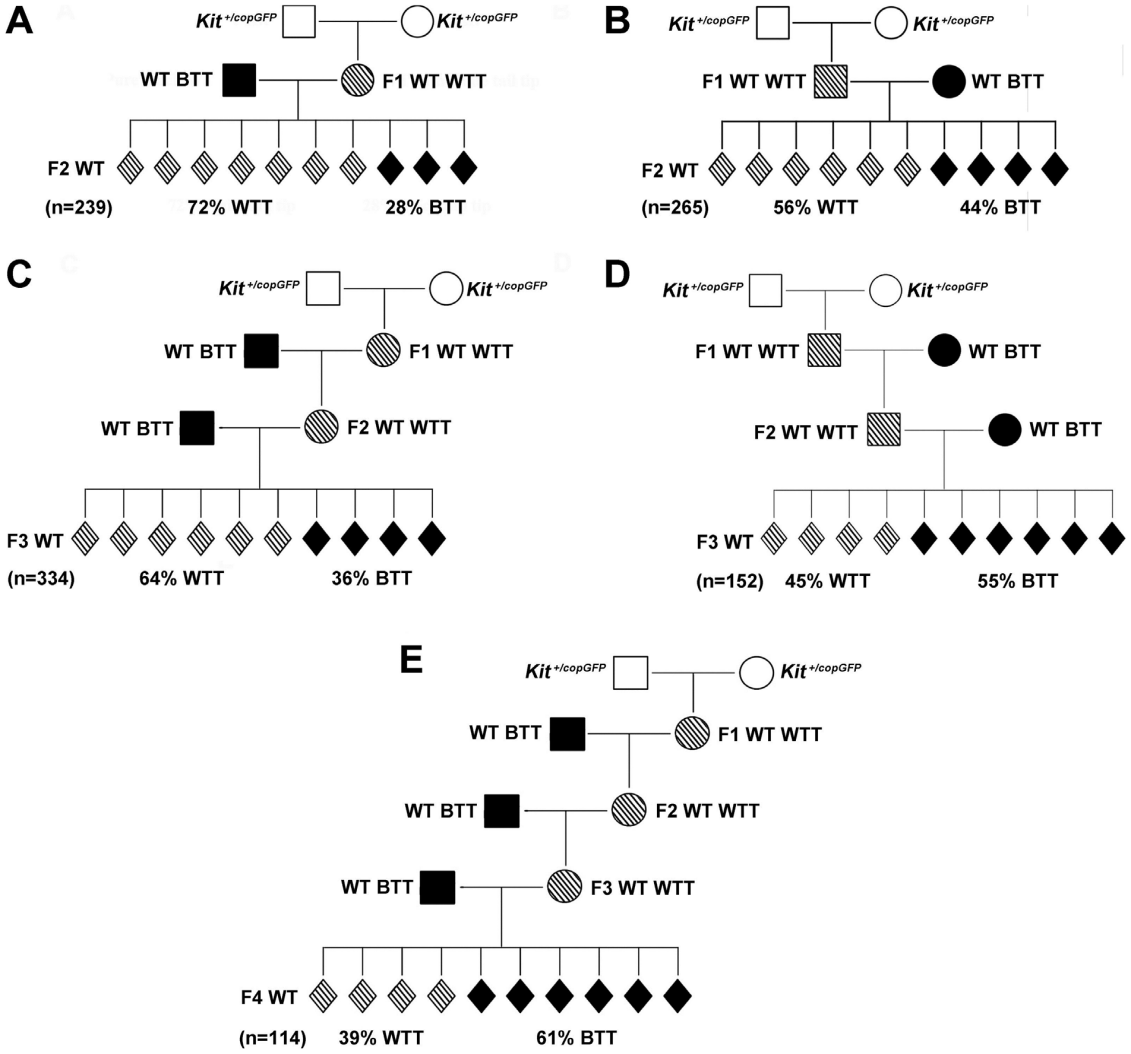
Transgenerational inheritance of the *Kit* paramutation phenotype in an outcrossing scheme. (A) Distribution of the WTT phenotype among WT F2s derived from WT WTT F1 mothers outcrossed with WT BTT fathers. (B) Distribution of the WTT phenotype among WT F2s derived from WT WTT F1 fathers outcrossed with WT BTT mothers. (C) Distribution of the WTT phenotype among WT F3s derived from WT WTT F2 mothers outcrossed with WT BTT fathers. (D) Distribution of the WTT phenotype among WT F3s derived from WT WTT F2 fathers outcrossed with WT BTT mothers. (E) Distribution of the WTT phenotype among WT F4s derived from WT WTT F3 mothers outcrossed with WT BTT fathers. “n” denotes the total number of offspring observed in each of the five mating schemes (A–E).

**Figure 4:**
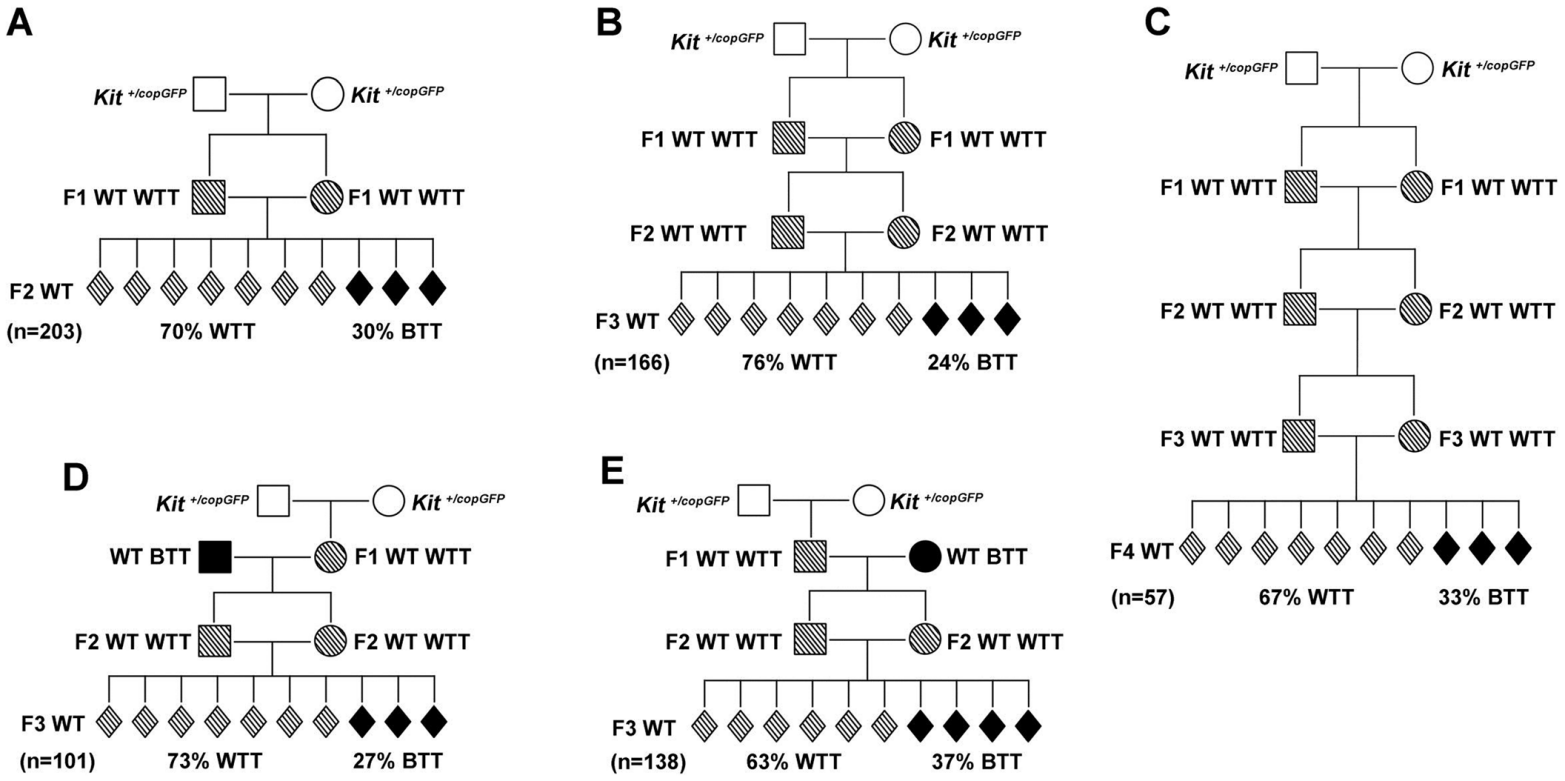
Transgenerational inheritance of the *Kit* paramutation phenotype in an intercrossing scheme. (A) Distribution of the “white-tail-tip” (WTT) phenotype among WT F2s derived from WT WTT F1 mothers intercrossed with WT WTT F1 fathers. (B) Distribution of the WTT phenotype among WT F3s derived from WT WTT F2 mothers intercrossed with WT WTT F2 fathers. (C) Distribution of the WTT phenotype among WT F4s derived from WT WTT F3 mothers intercrossed with WT WTT F3 fathers. (D) Distribution of the WTT phenotype among WT F3s derived from WT WTT F2 mothers intercrossed with WT WTT F2 fathers. Note that the F2 WT WTT parents were derived from outcrossing of WT WTT F1 mothers with WT BTT fathers. (E) Distribution of the WTT phenotype among F3s derived from WT WTT F2 mothers intercrossed with F2 WT WTT fathers. Note that the F2 WT WTT parents were derived from outcrossing of F1 WT WTT (1^st^ WT WTT) fathers with WT BTT mothers. “n” denotes the total number of offspring observed in each of the five mating schemes (A–E).

The paramutation phenotype, i.e., WTT, is present in F1 progeny of heterozygous parents (F0) with a penetrance of ~60% (Fig. 1D–F). However, it remains unknown whether the paramutation phenotype can be transmitted to subsequent generations. To determine the transgenerational inheritance of this novel *Kit* paramutation, we bred F1 paramutant mice using two breeding schemes: outcrossing and intercrossing. In the outcrossing scheme, we bred 1^st^ WT WTT mice with WT BTT mice that were totally unrelated to the *Kit* paramutation family for up to 4 generations (Fig. 3). In contrast, for the intercrossing scheme, 1^st^ WT WTT siblings derived from HET (*Kit^copGFP^*) parents were intercrossed to obtain F2s WT mice; F3 and F4 WT mice were obtained through intercrossing WT WTT F2 and F3 siblings, respectively (Fig. 4).

When 1^st^ WT WTT females were bred with WT BTT males, 72% of the offspring (F2s) displayed WTTs (Fig. 3A). However, when 1^st^ WT WTT paramutant males were bred with WT BTT females, only ~56% of the offspring (F2s) displayed WTT phenotype (Fig. 3B). The female germline (eggs) appears to transmit the paramutation with a higher efficiency, as compared to the male germline-mediated transmission in the 2^nd^ generation (72% *vs.* 56%; χ^2^ test, p < 0.01).

When F2 WT WTT females were further outcrossed with WT BTT males, ~64% of the F3 mice displayed WTTs (Fig. 3C), whereas WTTs were seen in only ~45% of the F3 mice derived from WT BTT females mated with F2 WT WTT males (Fig. 3D). The incidence of WTT was significantly decreased from F2 to F3 when the paramutation was transmitted through either the female (from 72% to 64%, χ^2^ test, p < 0.01) (Fig. 3A, C), or the male germline (from 56% to 45%, χ^2^ test, p < 0.01) (Fig. 3B, D), suggesting the paramutation phenotype is being “diluted” in subsequent generations in the outcrossing scheme. Since F3 mice derived from outcrossing of WT WTT males already displayed WTT levels close to the baseline in WT unrelated C57BL/6J populations (45% *vs.* 40%), we further outcrossed only the F3 WT WTT females with WT BTT males, which led to the baseline levels of the WTT phenotype (39%) (Fig. 3E).

The outcrossing scheme appeared to “dilute” the paramutation. To determine the outcome of intercrossing on transmission efficiency of the paramutation, we bred F1 WT WTT siblings derived from *Kit^copGFP^* heterozygous parents. F2 mice from the F1 intercrossing displayed an incidence of WTT at ~70% (Fig. 4A). When F2 WT WTT siblings were further intercrossed, ~76% of the WT F3s displayed the WTT phenotype (Fig. 4B). Similarly, the incidence of the WTT phenotype persisted at a similar rate (~67%) in WT F4s when the WT WTT F3 siblings were further intercrossed (Fig. 4C). Interestingly, when F2 mice derived from outcrossing of F1 WT WTT female were used for intercrossing, ~73% of the F3 WT mice displayed WTT (Fig. 4D). In contrast, when F2 mice from the outcrossing of F1 WTT-bearing males were intercrossed, only ~63% of the F3 mice had the WTT phenotype (Fig. 4E). The difference between the two intercrossing schemes was statistically significant, suggesting that “stricter” intercrossing between siblings derived initially from the heterozygous parents can maintain a higher transmission efficiency, whereas any outcrossing in between intercrossing would lead to a decrease in the transmission efficacy.

### Stochastic changes in levels of *Kit* coding and noncoding isoforms in mice carrying the paramutant allele

A paramutant allele usually displays altered gene expression profiles, which are often associated with a phenotype. Therefore, offspring that inherit the paramutant allele may display the phenotype in the absence of the paramutagenic allele (i.e., the copGFP allele in this study). To determine changes in *Kit* gene expression profiles, we conducted qPCR analyses on five tissues (brain, intestine, skin, testis, and ovary) collected from WT BTT (negative control for the *Kit* paramutation), WT WTT (*Kit* paramutation-unrelated, baseline WTT control), HET (mice with both the paramutagenic and the paramutant alleles), and 1^st^ WT WTT (mice carrying only the paramutant allele) mice. *Kit* can produce six transcript isoforms, among which only isoforms 1 and 2 encode KIT protein, whereas isoforms 3–6 do not contain ORFs and thus, are noncoding (based on ENSMUSG00000005672.8 in Ensembl Genome Browser). Since large noncoding RNAs derived from an mRNA-coding gene may affect the transcriptional activity of their host gene^33^,^34^, we examined the levels of all six *Kit* transcript isoforms using isoform-specific qPCR analyses (Fig. S1). Overall, levels of the protein-coding isoforms (isoforms 1 and 2) were not significantly altered in testis, ovary and brain samples collected from HET (*Kit^+/copGFP^*) and 1^st^ WT WTT mice, as compare to the controls, which were either WT WTT or WT BTT mice, both of which were totally unrelated to the *Kit* paramutation family. A minor, but statistically significant increase in levels of isoform 1 was observed in intestine between WT WTT and WT BTT mice (Fig. S1). Similarly, a moderate, but statistically significant decrease in isoform 2 levels was detected in the brain samples between WT WTT and 1^st^ WT WTT mice (Fig. S1). Levels of all four noncoding isoforms (isoforms 3–6) were highly variable among samples of three biological replicates, which may explain a lack of statistically significant differences among most of the five organs and four types of mice analyzed. However, a significant increase in levels of isoform 3 was detected in the ovary samples between 1^st^ WT WTT and WT BTT mice. In skin samples, isoform 4 levels were drastically decreased in HET mice, and levels of isoforms 4 and 5 were increased significantly in 1^st^ WT WTT mice, as compared to control (WT BTT) mice (Fig. S1). Overall, changes in all six *Kit* transcript isoforms appeared to be stochastic with a fairly high degree of variation among all five organs and all four types of mice analyzed. Together, these data suggest that the paramutant *Kit* allele, which is derived from either of the heterozygous parents, may display altered expression in both coding and noncoding isoforms of *Kit*, but the changes are likely subtle and stochastic in different tissues.

### No methylation changes in *Kit* promoter in sperm DNA, but changes occur in tail DNA samples

We then examined CpG methylation levels of the CpG-rich *Kit* promoter through bisulfite sequencing. We observed very low levels of CpG methylation in this region, with little variation across individual biological replicates from WT BTT, WT WTT, HET, and 1st WT WTT mice (Fig. S2A). Only one CpG was consistently methylated across all genotypes.

Sperm DNA possesses different methylation patterns, compared to DNA derived from somatic tissues^35^. However, it has been shown that many murine genes involved in zygotic development remain hypomethylated in sperm^35^. To determine whether this was the case for *Kit*, we assayed CpG methylation of the *Kit* promoter in tail DNA samples. Interestingly, we observed slightly higher levels of methylation throughout the *Kit* promoter region in WT BTT, WT WTT, and HET mice, whereas no methylation above the baseline was observed in 1^st^ WT WTT (Fig. S2B). However, these patterns were not entirely consistent between individual biological replicates, and were not strictly correlated with the presence of the paramutant allele in HET and 1^st^ WT WTT mice.

We next examined the ~1.5 Kb CpG-rich region surrounding the *Kit* promoter through MeDIP-qPCR (Fig. S3A). Both 5 mC and 5 hmC (5-hydroxymethylcytosine) methylation was examined in sperm DNA. 5 hmC modifications are associated with transient or dynamically regulated CpG methylation, and are often the intermediate products in the biochemical reactions that facilitate 5 mC removal or incorporation, and thus, the presence of 5 hmC modifications would be indicative of dynamic methylation regulation in this region^36^. We found no significant differences in DNA enrichment, which was calculated as percent enrichment of diluted input controls, among WT BTT (negative control for Kit paramutation), WT WTT (control for baseline WTT), HET (paramutant allele in the presence of paramutagenic allele) and 1^st^ WT WTT (paramutant allele in the absence of paramutagenic allele) mouse sperm (Fig. S3B, C). These data are consistent with our sperm DNA bisulfite sequencing data, supporting the idea that the *Kit* promoter is hypomethylated in sperm (Fig. S2). Taken together, we failed to detect significant changes in DNA CpG methylation patterns in the *Kit* promoter region that correlate with the WTT phenotype in mice carrying the paramutant allele. 

### Both paternal and maternal RNAs can induce the paramutation phenotype although it is non-heritable

**Figure 5:**
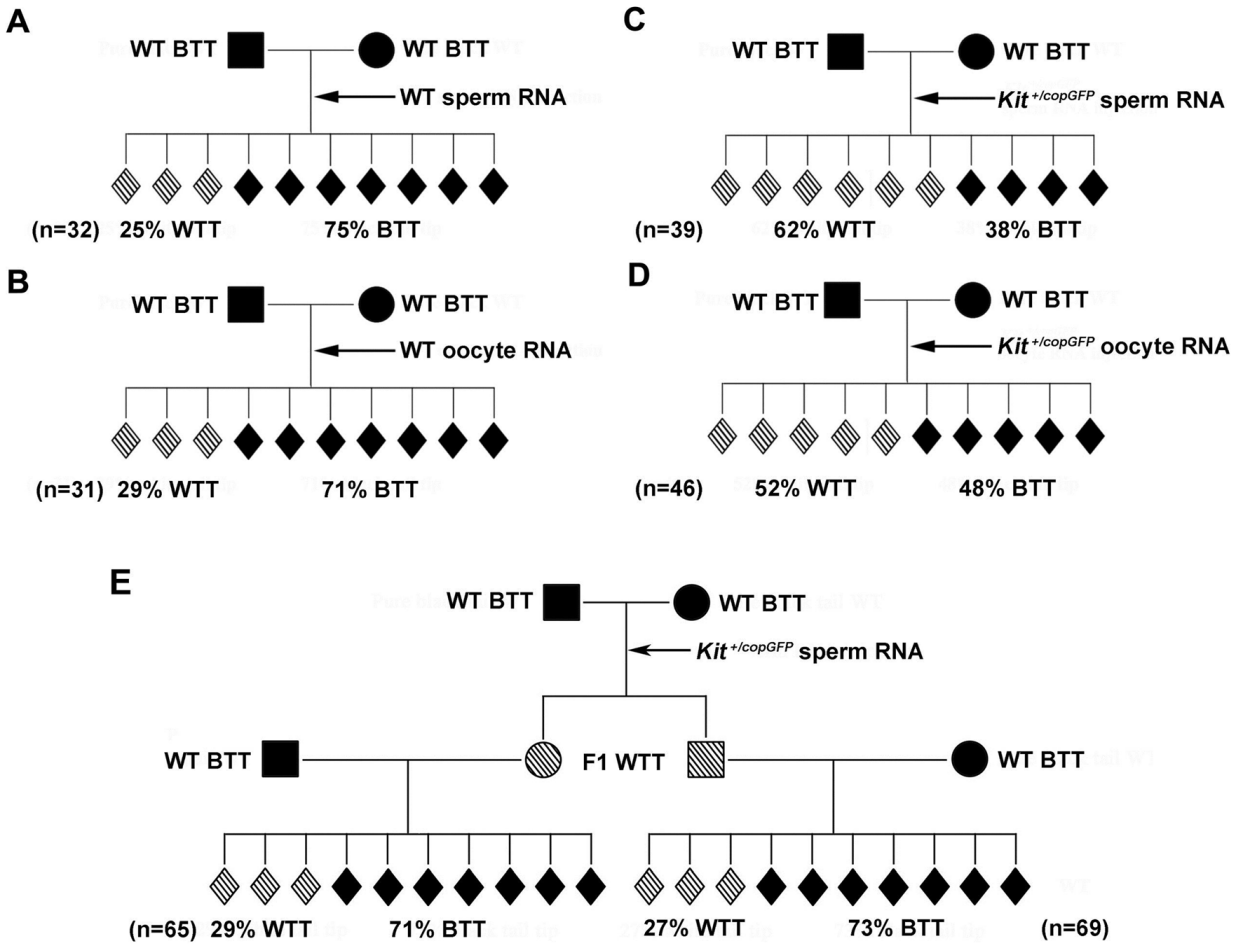
Injection of paternal (i.e., sperm-borne) and maternal (i.e., oocyte) total RNAs induced the “white-tail-tip” (WTT) phenotype. (A) Incidence of the WTT phenotype among offspring derived from WT BTT zygotes injected with WT BTT sperm-borne RNAs. Note that all mice used were WT BTT males or females. (B) Incidence of the WTT phenotype among offspring derived from WT BTT zygotes injected with WT BTT oocyte RNAs. Note that all mice used were WT BTT males or females. (C) Incidence of the WTT phenotype among offspring derived from WT BTT parents-derived zygotes injected with sperm-borne RNAs isolated from HET (*Kit^+/copGFP^*) males. (D) Incidence of the WTT phenotype among offspring derived from WT BTT parents-derived zygotes injected with oocyte RNAs isolated from HET (*Kit^+/copGFP^*) females. (E) Outcrossing F1 males or females carrying sperm-borne RNA-induced WTT phenotype with WT BTT females and males, respectively. “n” denotes the total number of offspring observed in each of the mating schemes (A–E).

It was reported that injection of RNAs isolated from *Kit^+/tm1Alf^* sperm into zygotes from mice unrelated to the *Kit^tm1Alf^* paramutant mice led to offspring with the WTT phenotype^12^. This finding suggests that RNA might be the mediator for paramutation establishment, and potentially its transmission through the germline. To test whether this finding applies to the *Kit^copGFP^* paramutation, we injected total RNA contents, including both small and large RNAs, isolated from not only sperm of HET (*Kit^+/copGFP^*) mice (as done in the previous study)^12^, but also oocytes, into the zygotes derived from WT BTT mating pairs that were totally unrelated to the *Kit^copGFP^* paramutant line. As a control, we injected paternal or maternal RNAs isolated from WT mice that were totally unrelated to the *Kit^copGFP^* paramutant line. Injection of either sperm-borne (i.e., paternal) or oocyte (maternal) RNAs isolated from WT, unrelated mice into unrelated WT zygotes led to close-to-baseline levels of WTT phenotype in offspring (25–29% vs. 30%) (Fig. 5A, B). However, injection of either paternal or maternal RNAs derived from *Kit^+/copGFP^* mice into unrelated WT zygotes resulted in offspring with an incidence of the WTT phenotype at between 52–62% (Fig. 5C, D), which is much higher than that in the control group and the baseline levels. Injection of sperm RNAs appeared to have a greater effect, as compared to injection of oocyte RNAs (62% vs. 52%), both of which were isolated from the HET (*Kit^+/copGFP^*) mice.

Both maternal and paternal RNAs appeared to be able to induce the WTT phenotype. To determine whether the induced paramutation phenotype could be transmitted to subsequent generations, we further outcrossed the WTT-positive, sperm RNA-induced mice (a total of 9 males and 15 females) with WT pure black C57BL/6J mice. To our surprise, offspring yielded by outcrossing of either male or female WTT-positive, sperm RNA-induced mice, displayed the baseline levels of the WTT phenotype (Fig. 5E). These data imply that although sperm-borne RNAs can induce the paramutation phenotype, the induced phenotype cannot be transmitted through the germline to subsequent generations.

### Maternal miRNAs and piRNAs affect the inheritance of the *Kit^copGFP^*-induced paramutation

DROSHA is a nuclear RNase III that cleaves primary miRNA transcripts into precursor miRNAs in the nucleus and thus, is essential for miRNA biogenesis^37^. Previous studies have demonstrated that oocyte-specific *Drosha* conditional knockout (*Zp3-Cre; Drosha^lox/−^*, hereafter called *Drosha* cKO) mice display normal oocyte development and fertility, although those *Drosha*-null oocytes contain neither precursor nor mature miRNAs^37^. *Mov10l1* encodes a protein that is required for PIWI-interacting RNA (piRNA) biogenesis, and global *Mov10l1* knockout mice do not produce piRNAs in any of their cells^38^,^39^. Therefore, these two KO lines provided an excellent opportunity to test whether maternal miRNAs or piRNAs could affect paramutation formation and transmission.

**Figure 6:**
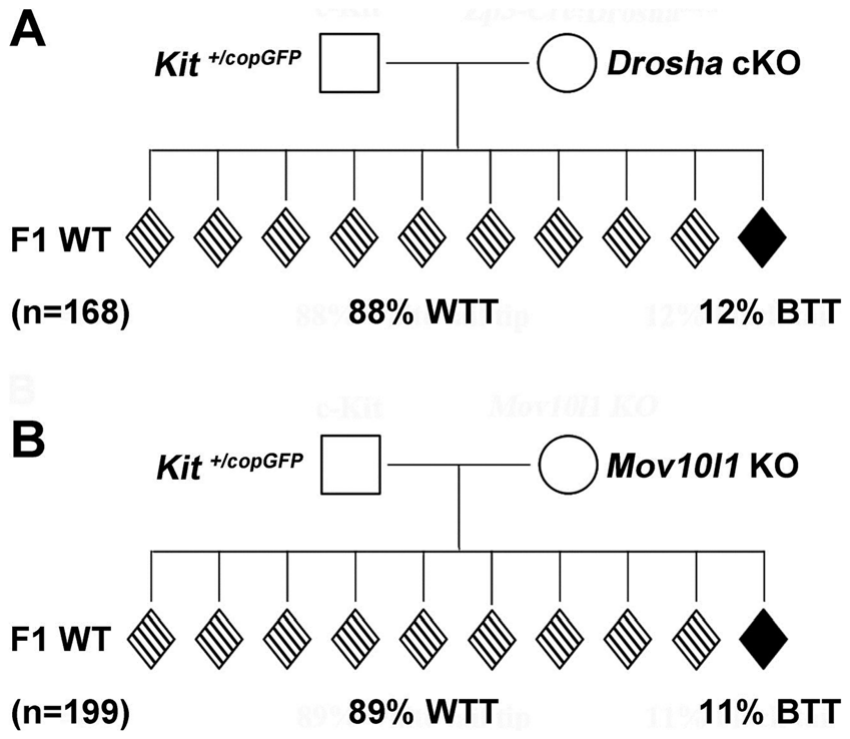
Effects of maternal miRNAs and piRNAs on the transmission of the “white-tail-tip” (WTT) phenotype. (A) Incidence of the WTT phenotype among offspring derived from HET (*Kit^+/copGFP^*) fathers and *Drosha cKO* mothers. “n” denotes the total number of offspring observed. (B) Incidence of the WTT phenotype among offspring derived from HET (*Kit^+/copGFP^*) fathers and *Mov10l1* KO (*Mov10l1^-/-^*) mothers. “n” denotes the total number of offspring observed.

We crossed *Kit^+/copGFP^* male mice with either *Zp3-Cre; Drosha^lox/Δ^* or *Mov10l1^-/-^* females, which were both on pure C57BL/6J background, and examined the WTT phenotype among all genotypically WT offspring (Fig. 6). When *Kit^+/copGFP^* male mice were bred with WT BTT, unrelated female mice, ~55% of offspring displayed WTTs (Fig. 1F). Interestingly, when *Kit^+/copGFP^* male mice were bred with *Drosha* cKO (pure black and miRNA-deficient in oocytes) and *Mov10l1* KO (pure black and piRNA-deficient in all cell types including oocytes) females, ~90% of offspring showed the WTT phenotype, which is significantly higher than the incidence seen when crossed with pure black WT females (55% *vs.* 88–89%, χ^2^ test, p < 0.01). Because the major difference between the pure black (WT BTT) females and the *Drosha* cKO or *Mov10l1* KO females lies in that the oocytes from the KO females contained no miRNAs or piRNAs, our data suggest that in normal oocytes, miRNAs and piRNAs can suppress the transmission of the *Kit* paramutation. Maternal miRNA or piRNA deficiency appears to enhance the transmission of the paramutation phenotype in this case.

## Discussion

Mutations in the *Kit* allele are often associated with coat color changes, e.g., mice carrying *Kit^w/wv^* allele are entirely white, *Kit^+/tm1Alf^* mice display white tail tips and white paws^12^,^32^. *Kit^+/CreERT2^* mice also display white bellies in addition to white paws and white tail tips^40^. We generated one null *Kit* allele, *Kit^copGFP^*, with the *copGFP* cassette inserted into exon 1 immediately after the start codon^31^. Heterozygotes all display white bellies, white paws and white tail tips^31^. The white spot phenotype results from the impaired *Kit* expression from the WT *kit* allele when the other is null^12^. Moreover, WT progeny of heterozygous parents tend to display WTTs, but without white bellies and white paws.

An earlier study has demonstrated that the WTT represents a phenotype associated with a paramutation induced by the *Kit^/tm1Alf^* mutation^26^. However, the validity of the study has been challenged because mice with WTTs are fairly common in most, if not all, of the lab mouse colonies of various strains^32^. In our own WT or transgenic mouse colonies, which are mostly on C57BL/6J or 129 Sv/Ev:C57BL/6J hybrid background, ~30% of the WT mice that are totally unrelated to the *Kit* mutant lines display WTTs, suggesting that the WTT phenotype is not unique to either the *Kit* genetic mutants or *Kit* paramutants. Despite the relatively common WTT phenotype in general lab mouse populations, it remains elusive how the WTT phenotype is formed. Nevertheless, the incidences of the WTT phenotype in general lab mouse populations represent baseline levels of this phenotype, and thus, should be defined so that the validity of using WTT as a phenotypic readout of the paramutation can be assessed more reliably. Our data suggest that the WTT can be used to evaluate the *Kit* paramutation effects because among WT progeny of *Kit^copGFP^* heterozygous parents, the WTT incidence is much higher than that in the general mouse population (30–40% in general population *vs.* 60–70% in WT offspring from *Kit^copGFP^* heterozygous parents). The elevated incidence in the WTT phenotype must be due to *Kit^copGFP^* –induced paramutation. It is of great importance to know the baseline incidence of the WTT phenotype because non-paramutant effects, otherwise, would have been taken into account, leading to potentially exaggerated conclusions. Equally important is that all analyses must be carried out using a large number of individual paramutant mice because some of them may be those with the baseline WTT phenotype, which is totally irrelevant to the *Kit* paramutation. However, when a large number of animals are analyzed, both *Kit* paramutation-specific and nonspecific WTT phenotypes will both be included, thus allowing for more reliable assessment of the paramutation effects.

Several lines of evidence suggest that the *Kit^copGFP^* paramutation is epigenetic by nature: First, the WTT phenotype exists in 60–70% of genetically WT mice derived from heterozygous parents, suggesting that the WT allele that they inherited from either or both of their parents has been modified, not in DNA sequence, but in functional status, and thus, represents the paramutant allele. Second, the WTT phenotype can be transmitted through the germline to subsequent generations; however, segregation of the phenotype does not follow the Mendelian Law, suggesting the modifications are not genetic, but epigenetic. Third, both maternal and paternal RNAs can induce the WTT phenotype in offspring of parents that are totally unrelated to the paramutation family, insinuating that the paramutation can be established by gamete RNAs during early embryonic development. Lastly, although the paramutation phenotype can be transmitted to subsequent generations, the penetrance decreases in later generations and eventually returns to the baseline levels after 3–4 generations when the paramutant mice are outcrossed. The transgenerational decrease in the penetrance of the paramutant phenotype most likely results from the global reprogramming events, which occur during preimplantation embryonic development and during PGC development. It has been shown that neither of the two global reprogramming events is complete because many imprinted loci and repetitive elements retain their epigenetic marks afterwards^29^. The persistence of the paramutation phenotype (i.e., WTT) across several generations, however, does suggest that the *Kit^copGFP^* paramutation is partially resistant to reprogramming.

In the outcrossing schemes, the male germline appears to be more capable of correcting the *Kit^copGFP^* paramutation because it takes less than three generations to bring the incidence of the paramutation phenotype down to the baseline levels when the *Kit* paramutation is transmitted through the male germline. In contrast, four generations are needed to do the same if the paramutation is transmitted through the female germline. This difference may result from the differential reprogramming mechanisms between the paternal and maternal genomes during the post-fertilization development, e.g., the former undergoes active demethylation, whereas passive demethylation occurs to the latter^29^,^41^. Therefore, a paternal paramutation may have a greater chance to be reprogramed than a maternal paramutation.

Compared to the outcrossing strategy, intercrossing appears to maintain the penetrance of the paramutation phenotype beyond four generations, if not indefinitely. The striking difference in penetrance of the paramutation phenotype between intercrossing and outcrossing schemes implies that a paramutation, or an epimutation in general, can persist in a population indefinitely in the case of intercrossing; it can be corrected and eventually “fade away” when outcrossed. The discovery that outcrossing leads to a dilution effect, while intercrossing causes persistence of the paramutation phenotype, is similar to phenotypes caused by genetic mutations. However, the distribution of the paramutation phenotype does not follow the Mendelian ratio, and can be explained by the developmental reprogramming. Nevertheless, this finding does imply that both outcrossing and intercrossing schemes should be evaluated in animal experiments on epigenetic transgenerational inheritance. Also, it suggests that in human populations, paramutations, or other types of epimutations, indeed can be corrected, or at least diluted during a period of several generations because human populations are largely outbred. However, paramutations, or epimutations in general, can persist for many more generations in a relatively inbred human population.

The previous study has demonstrated that injection of total RNAs isolated from *Kit^+/tm1Alf^* sperm to WT unrelated zygotes can induce the WTT phenotype, and this phenotype can be transmitted to the next generation^12^. Although not tested in the previous study, injection of maternal RNAs should yield the same results given that both male and female paramutant mice can transmit the WTT phenotype. Indeed, we demonstrate that both paternal (i.e., sperm-borne) and maternal (oocyte) total RNAs can induce the WTT phenotype when injected into WT, unrelated zygotes. These results further validate the notion that gamete RNAs can induce the *Kit^copGFP^* paramutation phenotype. However, the WTT phenotype fails to be transmitted to the next generation, which is contradictory to the previous study^27^. One possible explanation would be that we took the baseline WTT levels into consideration when drawing our conclusions. The failure in transmitting the germline RNA-induced paramutation phenotype suggests that gamete RNAs can induce the paramutation, but its stable transmission requires other factors. In other words, gamete RNAs may be sufficient to induce a paramutation, but are insufficient for the inheritance of the induced paramutation.

It would be critical to identify the factors essential for successful transmission of the gamete RNA-induced paramutation. A recent report demonstrates that maternal and early embryonic expression of DNMT2, a methyltransferase that mainly methylates tRNAs, is required for *Kit^tm1Alf^*-induced paramutation and miR-124-induced *Cdk2* paramutation^13^. However, one cannot determine, based on that study, whether the sperm-borne, methylated tRNA-derived small RNAs are required for the establishment or the transmission of paramutation. Moreover, it remains unknown whether the requirement for *Dnmt2* is a direct effect on germline sncRNAs, or an indirect effect on the stability of other factors essential for paramutation transmission.

Given that paramutation represents a special type of epimutation, the underlying mechanism for paramutation transgenerational inheritance could well be applicable to general epigenetic transgenerational inheritance. The finding that RNA injection into zygotes can induce a phenotype also suggests that although RNA is not generally considered genetic material, it can indeed alter the phenotype if introduced into early embryos. One can then imagine that supplementation of RNAs during IVF or ICSI in human fertility clinics could potentially cause phenotypic changes, although no DNA is introduced to the test-tube babies. One assuring thing, however, is that our data suggest that the RNA-induced phenotypic changes may not be transmittable to subsequent generations. Overall, RNA-induced phenotypic alterations may represent an ethical issue in reproductive medicine, although RNA is generally not considered genetic materials in general.

sncRNAs have been implicated in the establishment and transmission of paramutations^2^,^4^,^6^. However, miRNAs and piRNAs are both essential for spermatogenesis and a lack of either causes disrupted spermatogenesis, leading to no sperm or the production of defective sperm that cannot fertilize eggs^42^,^43^,^44^, thus precluding studies of the effects of sperm-borne sncRNAs on paramutations. However, a lack of either miRNAs or piRNAs in oocytes appears to be compatible with normal folliculogenesis and female fertility^37^,^39^, which provides an excellent opportunity for us to test the effects of maternal/oocyte miRNAs and piRNAs on the paramutation transmission. The significant increase in the incidence of the WTT phenotype (from 55% to 88–89%) in progeny of females with *Drosha*- or *Mov10l1*-null oocytes suggests that the maternal miRNA and piRNA pathways have a suppressive role in paramutation transmission. This finding implies that the maternal miRNA and piRNA machineries normally inhibit transmission of the paramutation, which could either be directly involved in sncRNA biogenesis during post-fertilization development, or acting indirectly on other factors essential for paramutation transmission through post-transcriptional or epigenetic regulations.

In summary, we report another paramutation mouse model, and demonstrate that the paramutation can be transmitted across multiple generations and the breeding scheme can drastically affect the transmission efficiency. Both paternal and maternal RNAs from paramutant mice can induce the paramutation phenotype, but effective transmission requires yet-to-be-defined additional factors. Whole genome/transcriptome approaches are needed to identify the molecular changes responsible for the establishment, maintenance, memory and transmission of the paramutation in the near future.

## Methods

### Use of mouse lines

All mice were maintained in a temperature and humidity-controlled, specific pathogen-free facility under a light-dark cycle (10 h-light/14 h-dark) with food and water *ad libitum*. Breeding and all experimental procedures were performed according to the mouse use protocols approved by the Institutional Animal Use and Care Committee (IACUC) of the University of Nevada, Reno.

*Kit^+/copGFP^* mice were generated as described in our previous report^31^. Zp3-Drosha cKO (*Zp3-Cre; Drosha^lox/−^*) and *Mov10l1* KO female mice were generated as described^37^,^45^. All three lines were backcrossed for at least 10 generations to the C57BL/6J background before used for the experiments reported here.

### Breeding scheme

Male and female *Kit^+/copGFP^* mice were bred to get the 1^st^ generation (F1) paramutant WT mice, and the number of F1 WTT-positive mice and the number of all F1s were recorded. In the “intercrossing” scheme, F1 WT WTT siblings were bred to obtain F2s; breeding of F2 WT WTT siblings led to the production of F3s; breeding of F3 WT WTT siblings led to the production of F4s, and the number of F2, F3 and F4 mice with the WTT phenotype was counted against the total number of F2s, F3s and F4s, respectively.

In the outcrossing strategy, F1 WT WTT males and females were bred with WT BTT, totally unrelated WT females and males, respectively, to obtain F2s. The number of WTT-positive F2 among the total number of F2s was determined. Similarly, F2 WT WTT males and females were further bred with totally unrelated WT BTT females and males, respectively, to obtain F3s, and the number of WTT-positive F3 among the total number of F3s was determined. F4s were obtained using the same outcrossing scheme.

In the mixed breeding scheme, WTT-positive F1s were outcrossed with unrelated WT BTT mice to obtain F2s. Subsequently, F2 WT WTT siblings were bred to yield F3s, and the number of WTT-positive F3 and the total number of F3s were then counted.

To test the effects of maternal miRNAs and piRNAs, *Kit^+/copGFP^* male mice were bred with Zp3-Drosha cKO and *Mov10l1* KO female mice, respectively. A trained observer determined the WTT phenotype based on the presence of the white spots on the tails, as illustrated in Fig. 1C.

### PCR-based genotyping

*Kit^+/copGFP^* mice can be genotyped using the primers as described^31^. Since these mice display white tail tips, white paws and white belly, *Kit^+/copGFP^* mice could be easily recognized.

### Preparation of sperm and oocytes

Caudal epididymal sperm were collected into HTF medium from *Kit^+/copGFP^* males. Followed by washing several times using PBS, the sperm pellet was stored in −80°C for subsequent analyses. For oocyte collection, 4–6 week-old *Kit^+/copGFP^* females were superovulated by intraperitoneal injection of 5IU of Pregnant Mare's Serum Gonadotropin (PMSG), followed by intraperitoneal injection of 5 IU of human Chorionic Gonadotropin (hCG) 48 h later. Mature oocytes were then collected from oviducts 14–16 h after hCG injection, and freed from cumulus cells by treatment in M2 medium containing 0.1% bovine testicular hyaluronidase. Followed by washing using the M2 medium, the oocytes were transferred into centrifuge tubes, and snap-frozen in liquid nitrogen for subsequent RNA extraction.

### RNA isolation

Total RNA was isolated from sperm, oocytes and different organs using the mirVana™ miRNA isolation kit (Ambion, Grand Island, NY) according to the manufacturer's instructions.

### RNA injection and embryo transfer

For collecting zygotes, WT BTT females (C57BL/6J) superovulated with 5 IU of PMSG (i.p.) followed by 5 IU of hCG (i.p.) 48 h later were mated with WT BTT males, and zygotes were collected by flushing the oviducts with M2 medium. For RNA injection, ~1–2 picolitre (pl) of total RNA (0.5 ng/μl) isolated from HET (*Kit^+/copGFP^*) or control (WT BTT) mouse sperm or oocytes were injected into the zygotes under inverted microscope using a microinjector (Cat# 930000043, Eppendorf). After injection, the survived embryos were transferred into oviducts of pseudopregnant CD1 (albino) females that had been mated with vasectomized males of the same strain during the night before. Cesarean section was performed on day 19 after embryo transfer to obtain live pups, which were then transferred to surrogate mothers.

### Bisulfite sequencing

Genomic DNA (gDNA) was isolated from either sperm or tail samples, using the GenElute Mammalian Genomic DNA MiniPrep Kit (Sigma). For sperm samples, the mirVana RNA extraction lysis buffer treatment was performed prior to this extraction (Life Technologies). After gDNA was isolated, 1 μg was bisulfite treated, following the instructions with the NEB Epimark Bisulfite Conversion Kit (NEB). Following cleanup, bisulfite-specific PCR amplicons were amplified using GoTaq 2X, covering the promoter region (Promega). These PCR products of 170 bp. were run on 2% agarose gels, and the bands of interest were isolated and gel purified with the Qiaquick PCR purification kit (Qiagen). The resulting DNA extracts were cloned into the pGEM T-easy subcloning vector (Promega), and 2 μl of ligation reaction was then transformed into 50 μl of 5α competent cells (NEB), and grown overnight on ampicillin-LB-Agar plates at 37C (NEB). Individual colonies were grown in ampicillin liquid culture and their DNA was extracted using the Zyppy plasmid Miniprep Kit (Zymo Research). Plasmid DNA was then digested with EcoRI (to confirm the insert) and sequenced, using the SP6 sequencing primer. Sequencing was performed at the Nevada Genomics Center. Individual CpGs were then quantified for bisulfite conversion and the totals were calculated.

### *Kit* isoform qPCR

RNA was isolated from the five tissues of interest, including brain, intestine, skin, testis and ovary, using the mirVana protocol (Life Technologies). RNA was reverse transcribed to obtain cDNAs, which were then diluted to 50 ng/μl and used for qPCR analyses, using FAST SYBR Green (Applied Biosystems). Each reaction contained 50 ng of cDNA and 1 μl of primer mix, in a total of 20 μl reactions. *Gapdh* and *Hprt* were used as endogenous controls. qPCR analyses were performed on a qPCR machine (7900HT Fast Real Time PCR System, Applied Biosystems). Relative quantification was performed with *Gapdh* serving as the endogenous control, and expression levels were further normalized against control wild type BTT samples. Primers used are listed in Table S1.

### *Kit* promoter MeDIP qPCR

Genomic DNA (gDNA) samples were prepared using the Mammalian Genomic DNA Miniprep Kit (Sigma). Eluted gDNA samples were sonicated to achieve DNA fragmentation with sizes ranging between 200–500 bp using the Bioruptor (Diagenode), with the high setting, with 3X (5X 0:30 On/0:30 Off) repetitions. DNA fragments were confirmed for their integrity by running ~5 μl on 2% agarose gels. For 5 mC IPs, an MeDIP Kit was used following the manufacturer's instructions (Active Motif, Cat. #55009). MeDIP products were subject to qPCR on a qPCR machine (7900HT Fast Real Time PCR System, Applied Biosystems). Primers used are listed in Table S1. Sample enrichment was relative to equivalent dilutions of sample input DNA, measured as percent enrichment. ΔCt values were obtained relative to the No Template Control (NTC) samples. For 5 hmC MeDIP, the same protocol was followed, but the 5 hmC antibody (Zymo, Cat. #A4001-25), was used in place of the 5 mC antibody, for the pull-down.

### Statistics

The χ^2^ test was used to evaluate differences in the frequency of the paramutation phenotype between the control and paramutant groups. The two-tailed student *t*-test was used to assess statistical significance for RT-qPCR analysis. Data were presented as mean ± SEM. Significance was set at p < 0.05 for all tests.

## Author Contributions

W.Y. conceived and designed the study; S.Y., D.O., A.S. and H.Z. performed the experiments; all participated in data analyses; W.Y. wrote the paper.

## Supplementary Material

Supplementary Information Supplementary Information
