# Supplementary material for: Breeding scheme and maternal small RNAs affect the efficiency of transgenerational inheritance of a paramutation in mice
Source: Sci Rep. 2015 Mar 18;5:9266. doi: 10.1038/srep09266 (PMC4363887; doi:10.1038/srep09266)
Supplement: Supplementary Information [file srep09266-s1.pdf]

Breeding scheme and maternal small RNAs affect the efficiency of transgenerational inheritance of a paramutation in mice

Shuiqiao Yuan[1], Daniel Oliver[1], Andrew Schuster[1], Huili Zheng[1] and Wei Yan[1,2,3]

[1]Department of Physiology and Cell Biology, University of Nevada School of Medicine, Reno, NV 89557, USA
[2]Department of Biology, University of Nevada, Reno, NV 89557, USA

[3]Co-corresponding authors:

Wei Yan M.D., Ph.D.
Professor
Department of Physiology and Cell Biology
University of Nevada School of Medicine
Center for Molecular Medicine, Room 207B
1664 North Virginia Street, MS 0575
Reno, NV 89557
Tel: 775 784 7765 (Office)
775 784 4688 (Lab)
Fax: 775 784 4362
Email: wyan@medicine.nevada.edu

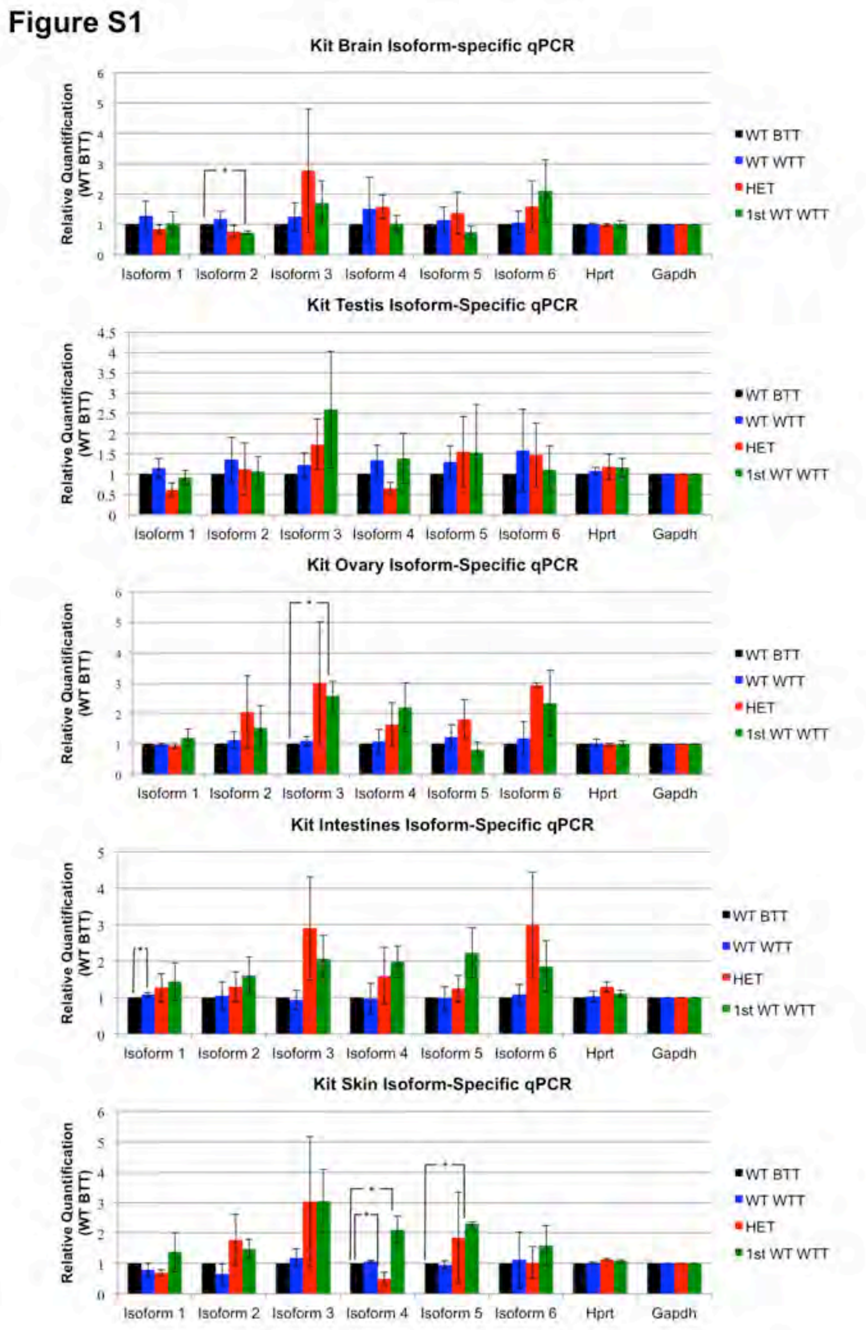

Figure S1. qPCR analyses of levels of six *Kit* isoforms in five tissues (brain, skin, testis, ovary, intestine) in control (WT BTT and WT WTT), and *Kit* paramutant (HET and 1st WT WTT) mice. Both *gapdh* and *Hprt* were used as endogenous control references, and the levels of the six isoforms were normalized relative to those in the WT BTT samples. Data are presented as means ± SEM. Biological triplicates were analyzed (n=3), and significant differences were determined using Student *t-test*, $p<0.05$.

Figure S2

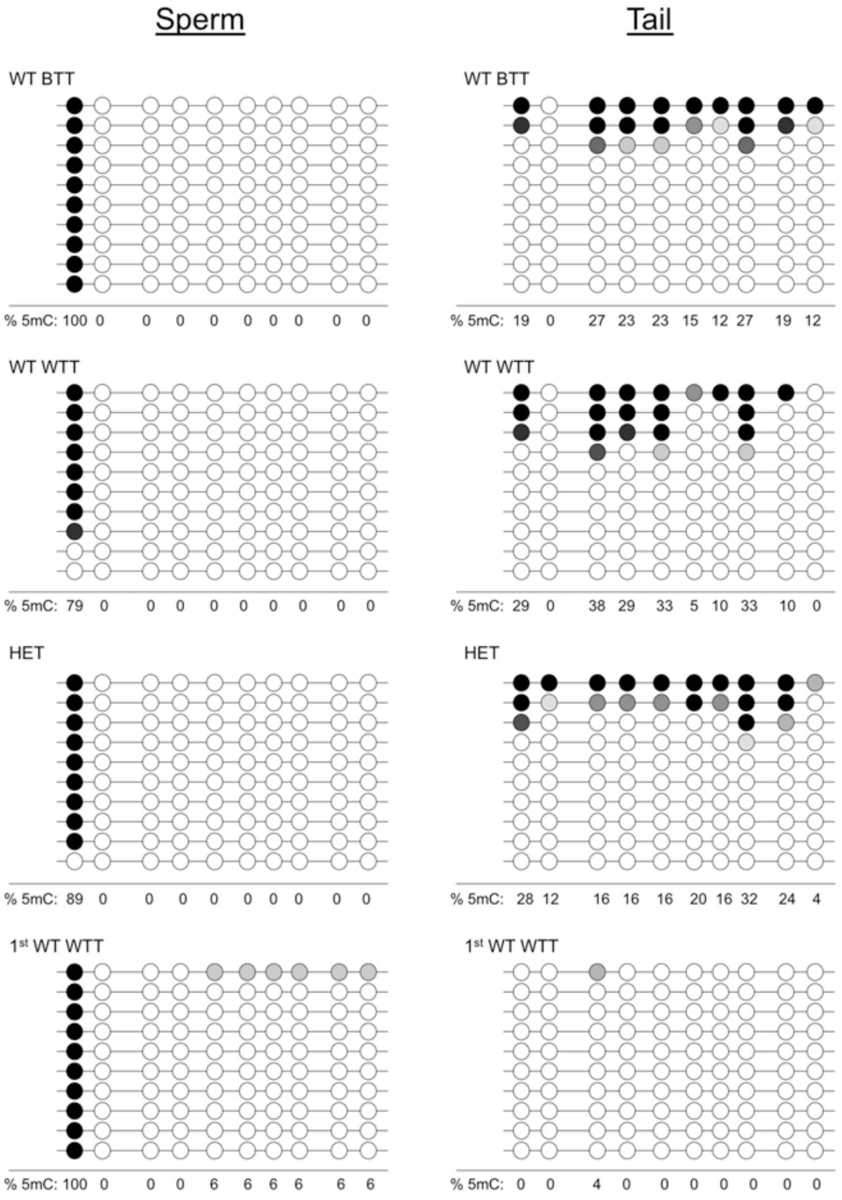

Figure S2. CpG methylation status of the *Kit* promoter regions in sperm (A) and tail (B) DNA of controls (WT BTT and WT WTT), HET (*Kit*$^{+/copGFP}$) and F1 WT WTT (1st WT WTT) mice, as determined by bisulfite sequencing assays. Vertical columns stand for individual CpGs that are present in this region (10 in total). Each row represents 10% of the clones sequenced. Black dots denote full 100% 5mC, whereas white dots indicate 0%. Gray dots represent 5mC levels in between these two extremes. Average percentages are shown below each panel. Ten clones from biological triplicates were analyzed for each sample set.

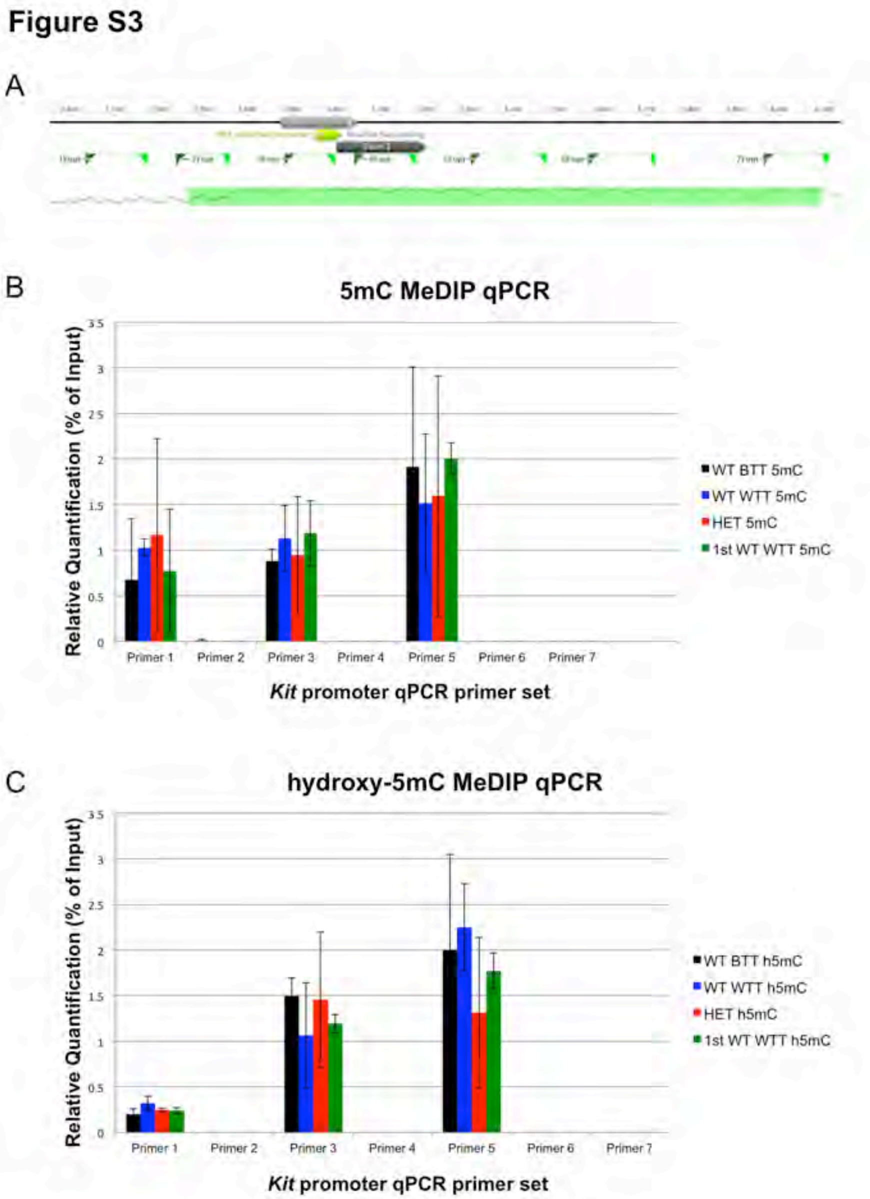

Figure S3. Levels of 5mC and hydroxyl-5mC methylation in sperm DNA of WT BTT, WT WTT, HET (*Kit*$^{+/copGFP}$), and 1st WT WTT (F1 WT WTT) mice. (A) A schematic diagram of the promoter region of the *Kit* gene. The EPD-predicted promoter region is depicted as a lime green box, and the region that was analyzed using bisulfite sequencing is shown as a light grey box (denoted "Bisulfite Sequencing"), and the primer pairs used in MeDIP-qPCR detection are indicated by the dark green (forward primer) and light green (reverse primer) pairs, 1-7, respectively. (B) 5mC-MeDIP qPCR analyses on sperm DNA. Primer pairs 1, 3, and 5 showed enrichment of DNA in the MeDIP products, relative to equivalent levels of input DNA control. No significant difference was detected between WT BTT samples and any of the three other genotypes. (C) h5mC-MeDIP qPCR analyses on sperm DNA from the four genotypes. No significant differences were observed between the four genotypes.

Table S1. Oligonucleotides used in qPCR and bisulfite sequencing analyses.

qPCR primer set:	Fwd Oligo (5'-3')	Rev Oligo (5'-3')
MeDIP-Opt1	CACGCTGGCCAATAGAAATCA	TGTTGGTAGAGGAGGACGGAT
MeDIP-Opt2	CACCTCCACCATAAGCCGAAT	ACGAGCGCATTAGGTACGG
MeDIP-Opt3	CTCCTGGTCTTAGAGGGCACA	CCACGCCCCTCCCTTCTCC
MeDIP-Opt4	CTAGTGGCTCTGGGGGCTC	AACAGGACGCAGAGCAGATC
MeDIP-Opt5	TTTTCCCATGCCTACCACCTC	AAAGCATCACCAAACTCGCC
MeDIP-Opt6	TTGTTAAAAGTTGCGCGTGGG	CAGTTGCGTCCCCAGCACT
MeDIP-Opt7	GTGTTCGTTGCTGCCACGC	CAAAGCTGGGGAGAACTGACT
Coding Isoforms (1&2)	AGACAGCCACGTCTCAGCCAT	AGTCCATCTGACAAAGTCGGG
Isoform 1	CTGGGAGGAGGGCTGGAGG	CGCTAGACTCTGAGCTCCTCT
Isoform 2	AACTGGTGGTTCAGAGTTCCA	GGCCTGGATTTGCTCTTTGTT
Isoform 3	GAGGCGGGATTATCTAAGGTA	CGAGTCACGCTTCCTTCTCAA
Isoform 4	ACCCTGCCTTCTTCCTTCCTA	AAATGGGCACTTGGTTTGAGC
Isoform 5	CTGGACAACATACCCACTGCA	TGCAGGAAGAGAAATAGCCTCA
Isoform 6	TACACCCTCTGCTTTGGCTTT	CAGTCCTTCCTTCACAGTGGG
Gapdh Control	AACTTTGGCATTGTGGAAGG	CACATTGGGGGTAGGAACAC
Hprt Control	TCAGTCAACGGGGGACATAAA	GGGGCTGTACTGCTTAACCAG
Kit promoter bisulfite PCR	TTGTGGGGGTTTTTGGTTTTAGAG	CACTAACGACGCGCGACAAC
